# IBM-XT processing of selected ion monitoring, data files from a Hewlett-Packard 5970 mass selective detector

**DOI:** 10.1155/S1463924687000324

**Published:** 1987

**Authors:** Herman Valente

**Affiliations:** Wadsworth Center for Laboratories and Research New York State Department of Health Empire State Plaza Albany New York 12201 USA


Journal of Automatic Chemistry/*Journal of Clinical Laboratory Automation*, Vol. 9, No. 4 (October–December 1987), pp. 158–165

### IBM-XT processing of selected ion monitoring data files from a Hewlett-Packard 5970 mass selective detector

**Herman Valente**

*Wadsworth Center for Laboratories and Research, New York State Department of Health, Empire State Plaza, Albany, New York 12201, USA*

*Data files from selected ion monitoring (SIM) acquisitions by a Hewlett-Packard GC/MSD system, were transferred to and processed by an IBM-XT personal computer. Using a series of programs written in the author's laboratory (TASQ, Target Analyte Search and Quantitation), a processing scheme was implemented in order to routinely quantitate polychlorinated dibenzo-p-dioxins (PCDDs) and polychlorinated dibenzofurans (PCDFs) in environmental samples. As a result, samples can be analysed with greater speed and results can be analysed and reported with greater flexibility than was previously possible. The technical details for transferring data files from the Hewlett-Packard system to the IBM-XT, as well as the programs that process these files, are discussed.*

### Introduction

The considerable number of environmental samples analysed in the author's laboratory, and the wealth of information typically generated by Gas Chromatography/Mass Spectrometry (GC/MS) techniques, have focused interest in applying the powerful features of today's desk-top microcomputers to the needs of environmental analysis [1 and 2].

When the laboratory became involved with the routine analysis of polychlorinated dibenzofurans and polychlorinated dibenzo-p-dioxins (PCDFs and PCDDs, respectively), it was realized that processing the large amount of acquired data would require designing and implementing a fast and reliable data processing scheme. Such a 'scheme' would have to quantitate PCDFs and PCDDs from GC/MS data by (1) searching Selected Ion Monitoring (SIM) data files in order to identify potential quantitation targets; (2) comparing tentatively identified peaks to those present in a SIM data file obtained from a standard sample; and (3) producing a quantitation report.

This paper describes the technical details for transferring data files from a Hewlett-Packard 9144 'QuickSilver' workstation – part of a Hewlett-Packard (Model HP 5970 MSD) gas chromatograph/mass selective detector system – to an IBM XT personal computer (PC). A set of interacting compiled BASIC programs, that were written in this laboratory, are also discussed. These programs process SIM data files to produce a quantitation report with greater speed and flexibility than would otherwise be possible using only vendor-supplied software. For convenience, these programs are collectively referred to as TASQ programs; an acronym for Target Analyte Search and Quantitation.

### Background

Routine analysis of environmental samples by Gas Chromatography/Mass Spectrometry has existed for more than a decade [3]. Prompted by a surge of public environmental awareness, this branch of analytical chemistry has enjoyed sustained technical growth. At present, nearly all federal, state and private laboratories that provide environmental testing services use some form of GC/MS to perform a vital part of their work.

When GC/MS techniques are used to analyse environmental samples, it very often leads to the challenge of processing the large amount of data produced. Fortunately, major advances in the semiconductor industry have placed unprecedented data-processing power in the hands of the analytical chemist, particularly since the first desk-top computers appeared in the late 1970s. These technical innovations have been an invaluable aid to data processing. In addition, software developed by both vendors of GC/MS systems and independent programmers have helped to keep pace with the demands imposed by environmental analysis. Driven by this progress, the field of environmental analysis continues to grow in new directions. Furthermore, as new toxicologically potent chemicals are discovered, new sources of contamination are found, and the effects of long-term exposure are unveiled, there is a concurrent demand for greater accuracy, expediency and thoroughness of analysis. As a result, environmental laboratories must constantly update the methods by which they handle samples, process data and report results.

### Discussion

To further this laboratory's ability to routinely analyse environmental samples for polychlorinated dioxins and furans, a Hewlett-Packard (HP) model 5970B Mass Selective Detector interfaced with an HP 5890A Gas Chromatograph, and an HP 9144 workstation, were purchased. The workstation is a stand-alone dedicated computer that controls nearly every instrument parameter on both the gas chromatograph and mass spectrometer. It also contains the vendor-supplied software that sets up the system for data acquisition, data processing, quantitation reports, etc. The gas chromatograph is equipped with a 30 m DB-5 capillary column (0·2 mm i.d., 0·33 μm film thickness) for high resolution gas chromatography (HRGC), and the mass spectrometer operates at nominal mass resolution (low resolution mass spectrometry, LRMS). The latter is also capable of selectively monitoring a user-specified group of ions (SIM). The SIM mode effectively increases the instrument's sensitivity when compared to full scan acquisitions [4].

The high stability of the mass spectrometer, the low maintenance and down-time of the system, and the quality of the data obtained made this hardware arrangement suitable for routine analysis. However, despite the impressive combination of features included with the workstation's software, new data processing methods were sought to increase productivity and broaden the scope of quantitation (see below). To understand how the data processing scheme currently in use was conceived, it is necessary to assess some of the limitations imposed by processing data through the workstation and by using the 'target compound quantitation' method.

When data acquisition ends, the resulting raw data file (ion chromatogram) is integrated by system-resident software to produce an integrated data file. In the SIM mode, this file contains the peak intensity and retention time of every eluted component with a nominal mass preselected by the operator (approximately 80 masses for dioxins, furans and their isotopically labelled analogues). The integrated data file is subsequently processed by the system's software to produce a quantitation report. This approach presents two main disadvantages:

(1) The workstation is a single task system, consequently the processing of integrated data files through system-resident software takes up valuable time that could otherwise be used to acquire more raw data.

(2) To discriminate extraneous signals from those of potential quantitation targets, the system-resident software compares the retention times of peaks within a sample to those of labelled internal standards, or external standards. Thus, the ability to identify targets is limited by the availability of the corresponding standards. This method of quantitation is known as a 'target compound quantitation' and can seriously limit PCDF and PCDD analysis. While there are 75 possible isomers of PCDD and 135 possible isomers of PCDF, only a fraction of these are available as labelled or unlabelled standards. The lack of standards is one of the most serious limitations afflicting this type of environmental analysis. To quantitate PCDDs and PCDFs for which there are no standards, it is necessary to process the data manually. At best, this is a time-consuming and error-prone solution. For example, processing a single data file to identify all possible tetrachlorodibenzofurans (TCDFs, 38 isomers) would entail a search for retention time matches between peaks of mass 306 and mass 304, and comparing the intensity ratios (intensity of mass 306/intensity of mass 304) of the resulting matching pairs to the theoretical ratio for this group of isomers (1·30, based on naturally occurring Cl isotope abundances). The total number of retention time and intensity ratio comparisons required varies from sample to sample, depending on the degree of contamination, noise levels, etc. Usually 60 to 120 comparisons are needed for TCDFs alone. To identify all dioxins and furans by chlorination level in one sample would require between 500 and 1500 comparisons. Afterwards, retention time comparisons between samples and standards, and the calculation of absolute sensitivities and analyte concentrations, would all have to be performed by hand to obtain a final quantitation report. Obviously, the number of mathematical manipulations in these steps can be staggering, while the potential for human error is high.

To overcome these limitations it was decided to investigate possible ways in which integrated data files could be transferred to and processed by an IBM XT personal computer (PC). This new approach would allow dedicating the GC/MS system to nearly full-time data acquisition, and, in addition, it would place integrated data files into the highly flexible processing environment of a desk-top microcomputer.

Mass 305.90 amu

| Ret Time | Type | Area | Height | Area % | Ratio % |
|---|---|---|---|---|---|
| 35.935 | BV | 385496 | 3308 | 18.168 | 100.00 |
| 36.457 | VV | 233493 | 1466 | 11.005 | 60.57 |
| 38.200 | PV | 182163 | 1316 | 8.585 | 47.25 |
| 38.504 | VV | 124164 | 1262 | 5.852 | 32.21 |

Mass 303.90 amu

| Ret Time | Type | Area | Height | Area % | Ratio % |
|---|---|---|---|---|---|
| 35.936 | VV | 286312 | 2614 | 22.069 | 100.00 |
| 36.489 | VV | 170587 | 1073 | 13.149 | 59.58 |
| 38.172 | PV | 57489 | 947 | 4.431 | 20.08 |

*Figure 1. Part of an HP generated integrated data file.*

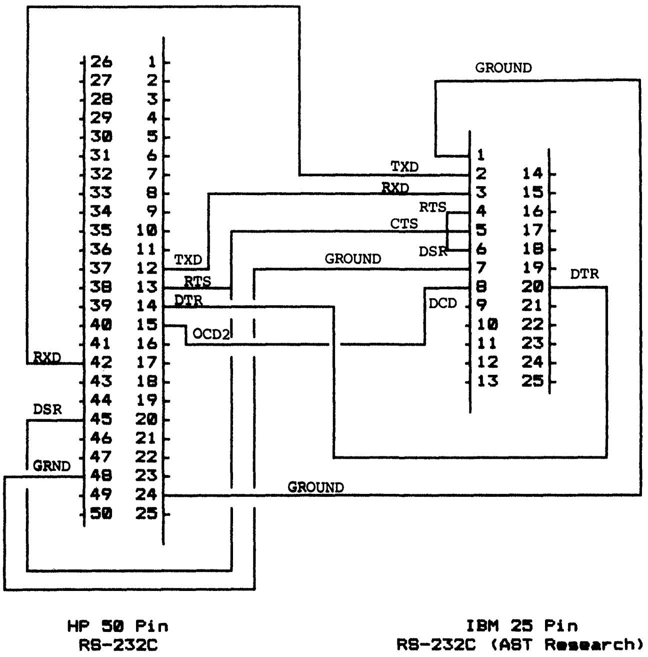

*Figure 2. Hewlett-Packard to IBM XT serial port connection.*

The following is an account of how the successful transfer of integrated data files, and the use of customized software written by the author, have allowed processing data files from a considerably larger number of samples in a shorter period of time, while eliminating the need for manual processing of data.

*Data transfer*

As discussed earlier, an integrated data file contains the intensity and retention time of every peak in the ion chromatogram. The content of this file can be automatically arranged and printed into several formats. The 'Area Percent' format can be sorted in two different ways: (1) it can be a single list in which the intensities of eluting peaks, regardless of their nominal mass, are listed in ascending order of retention time; or (2) several lists, one per nominal mass, with all intensities listed in ascending order of retention time. Files arranged in the latter format are routinely transferred to the PC because they are more readily processed for reasons that will become clear later. Figure 1 partially illustrates the appearance of these files.

The transfer of integrated data files from the workstation to the PC is carried out through their RS-232 serial ports; a standard feature on both systems. The port to port hardwire diagram is shown in figure 2. The procedure also requires some type of communications software for the PC and the report generator software that is provided with the GC/MS system. For convenience, all files are transferred in ASCII format [5]. Since integrated data files are not in this format, they must be translated by the HP workstation, and the resulting file saved on disk. To effect the transfer of data using the ASCII file as the input file, it is necessary to use the 'Pascal Filer' available from the vendor. The Filer can perform a number of file operations, one of which transfers ASCII files to a printer. When this option is selected, the Filer reads the data file and then sends the output to the printer port. In this case, the port used by the Filer is the 50-pin RS-232C jack behind the workstation. This connector is used to link the HP system to the PC. The communications software allows the PC to receive the data files. There are a number of software packages that can be used for this purpose. KERMIT version 2·29 (or higher) works very well at a baud rate of 4800 [6]. Once transferred, the data file is ready to be processed by the microcomputer.

*TASQ programs*

The ASCII file transferred from the HP system is a duplicate of the integration file. Thus, it is arranged as illustrated in figure 1. To process this file it was necessary to write a series of programs containing the routines that search the data and follow the decision-making criteria by which PCDDs and PCDFs are found and quantitated. These programs were written in BASIC language due to its powerful string manipulation capabilities, and the high portability and processing speed of the compiled programs. Many sub-routines were added to speed up processing time by avoiding unnecessary reading of files and data entry through the keyboard. To date, 14 compiled BASIC programs have been written. Three of these programs perform the three fundamental processing routines: (1) search data files for peaks that qualify as likely targets; (2) compare the peaks selected during the first step with data files generated from standard samples; and (3) quantitate and report all positively or, if desired, tentatively identified peaks. The programs are called Automatic Peak Matching (APM), Retention Time Matching (RTM) and Automatic Quantitation Report (AQR), respectively. How they operate will be discussed in some detail. The other 11 TASQ programs create files needed by the first three programs, produce a variety of utility reports, or create files that are compatible with commercially available software such as Lotus 123 [7] and Sideways [8]. These will be discussed briefly. Figure 3 shows the scheme through which data files are transferred and processed.

*Automatic peak matching*

The criteria used to tentatively identify targets are applied by APM. It is the only TASQ program that processes data files transferred from the HP workstation. APM contains several string and data file manipulation routines that are adjusted to the features common to all integrated data files. Thus, for example, it can find the integration list for mass 306 by searching for the string 'Mass' and reading the rounded out number appearing next to the string. Intensities and retention times are read from the file by searching the unique locations at which these values appear relative to the string (see figure 1). As each value is read, it is placed into a list until the end of the table is reached. This results in two lists, one for retention time and another for intensity. Although the file may contain more tables than needed, only those for masses appearing in a 'Target List File' (*.TLF, where (*) is the filename and 'TLF' is the filename extension) are used. *.TLF files are created by the Target List Editor (TLE) program. APM uses this file to read the searching parameters needed to find potential targets. It lists the names, number of possible isomers, theoretical intensity ratio, quantitation ion mass, ratio ion mass, percentage retention time tolerance and percent ratio tolerance for every target. Two mass tables from the data file are needed per target. Thus, four lists are compared to extract qualifying peaks. The comparison is called a 'Peak Matching' routine (PM). Figure 4 shows how the lists and the *.TLF file data are used for this purpose.

As targets are identified, results are stored in an 'Intensity Retention Time' file (*.IRT). This file contains the quantitation ion intensity and retention time of every qualifying peak, and the target name for which they qualify. The data in this file is used in the next processing stage. It should be noted that all TASQ programs rely on a target naming system to properly carry out many of their basic functions. This system will be described later.

*Retention time matching*

To positively identify a target it is necessary to match the retention times in the *.IRT file with the retention times of standards. Two methods are used for this purpose. Internal Retention Time Matching (I-RTM) compares the retention times of native targets with those of labelled internal standards. External Retention Time Matching (E-RTM) uses the retention times of both labelled and unlabelled standards from a second *.IRT file generated

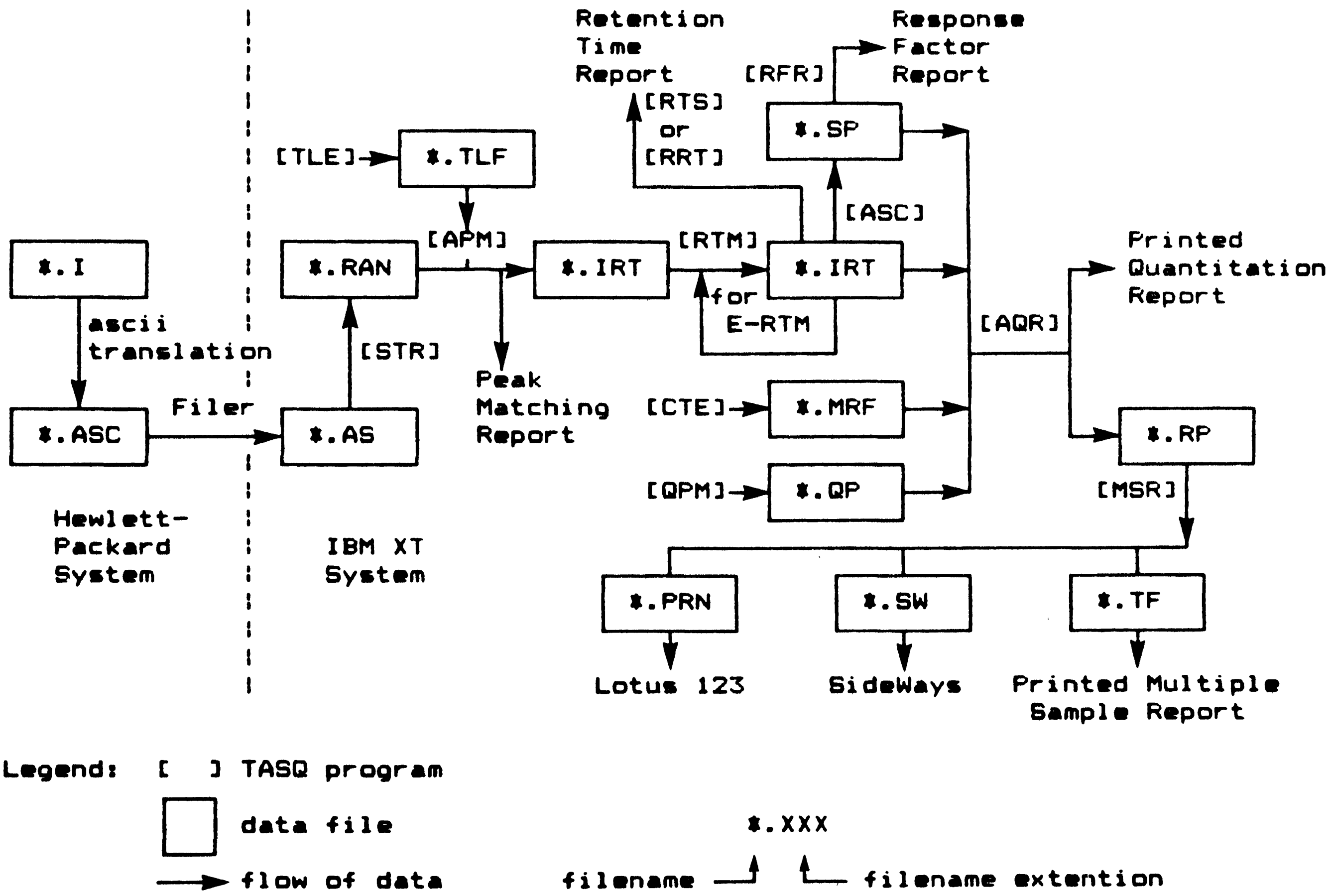

*Figure 3. Data transfer and processing scheme.*

by a standard sample. The RTM program makes both possible. It utilizes a *.IRT file(s) created by APM and can be used as an editor. RTM's retention time matching routines are identical to those use by APM. During I-RTM two retention time lists are created; one for native peaks and another for internal standards. When the retention time of a standard matches that of a native within a specified tolerance, RTM automatically replaces the target name based on the name of the standard peak. To accomplish this, a very simple yet effective naming system is used. Under this system there are two levels of characterization a target can attain: generic and specific. There are also two types of specific names: one for specifically identified natives or unlabelled standards; and another for specifically identified labelled standards. Generically identified peaks are those that have only met the identification criteria used by the APM program. Specifically identified peaks have, in addition, met the retention time matching criteria set by the RTM program. The name of any given target may contain one to three identifiers. The generic labelling – specific – generic identifier is always present, while the specific and labelling identifier may, or may not, be. When a name contains more than one identifier, it is hyphenated between identifiers. This naming format easily allows a TASQ program to recognize the level of characterization a target has reached. When a peak's name has no hyphenation it has attained the lowest level of characterization. Those with any hyphenation at all are specifically identified as an unlabelled standard or native (one hyphen) or as a labelled standard (two hyphens). The identifier's nomenclature is determined by the user when a *.TLF file is created. In the case of PCDDs and PCDFs, the generic identifiers used describe the chlorination level and whether it is a dioxin or furan, i.e. TCDD, TCDF, etc. The specific identifiers indicate the chlorine substitution pattern, i.e. 2378, 12378, 12346789, etc. Labelling identifiers are less informative. Carbon-13 and chlorine-37 labelled standards use 'C13' and 'CL37' identifiers, respectively. For obvious reasons, hyphens are never used within an identifier. When a *.TLF is created, except for labelled and unlabelled standards, names without hypens are used.

The naming systems describes above enables RTM to rename peaks when a retention time matching condition exists. When this occurs, the specific identifier portion of the standard's name is added to the matching target name. Thus, if a target named 'TCDD' matches the internal standard peak named C13-2378-TCDD, the former name is changed to '2378-TCDD'. This system also supports several time-saving logical operations, such as not comparing the retention times of a native and standard peak with different generic identifiers, etc.

A correction factor is applied during E-RTM. Small differences in oven temperature, carrier gas pressure, and other instrument conditions, can cause retention times to shift from one sample to the next. The magnitude of this effect is often large enough to preclude proper retention

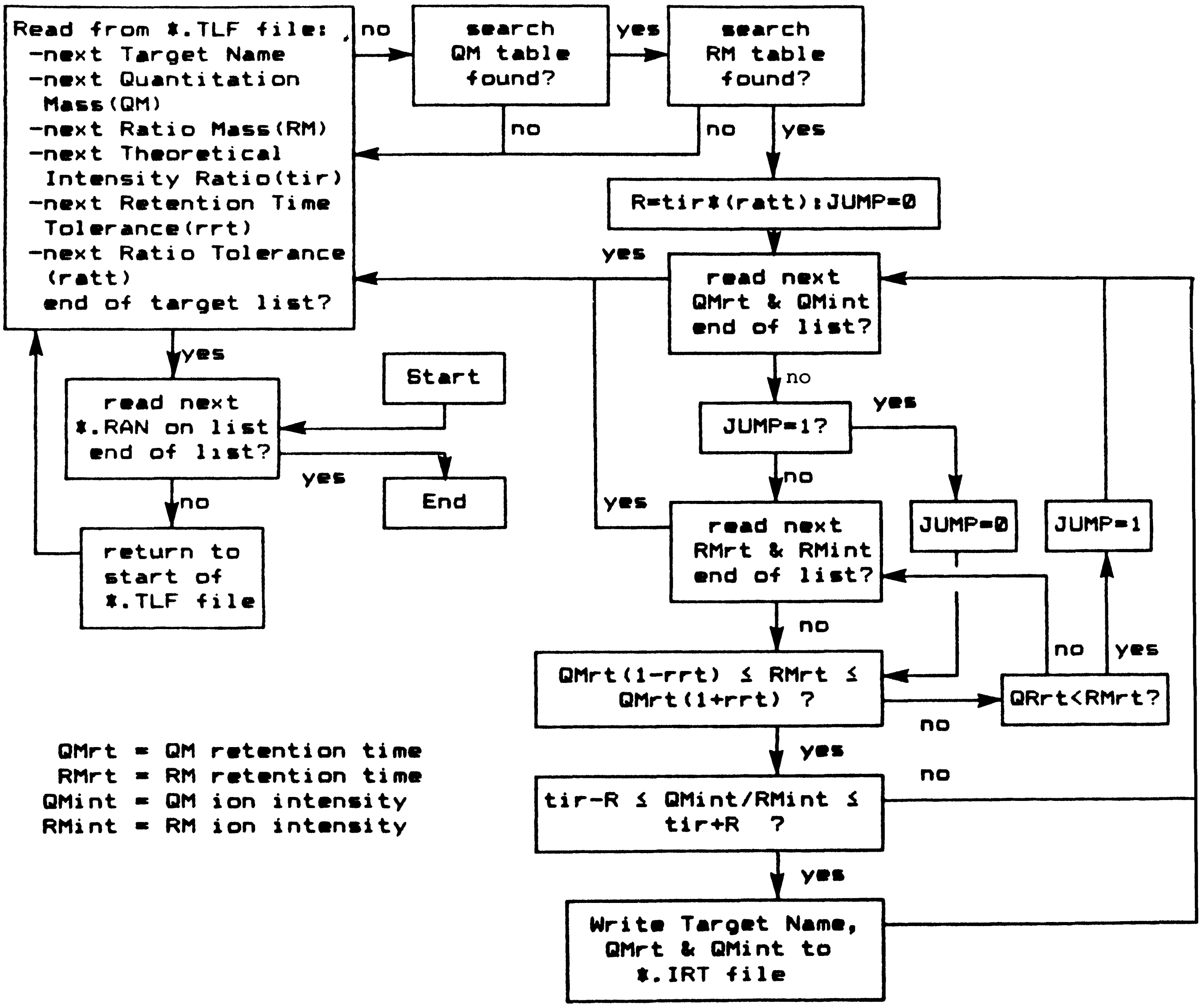

*Figure 4. Peak matching routine used by APM.*

time comparisons, or more seriously, it can lead to the erroneous identification of native targets. To eliminate this effect, an internal standard common to both *.IRT files is selected for use as a retention time surrogate standard. The difference in retention times for this standard is then used to mathematically offset the retention times listed in the *.IRT file of the standard sample before the E-RTM process takes place.

Another RTM feature allows totalling the intensities of targets with the same generic identifier. This routine creates new entries with 'TOT' as specific identifiers. As a result, names such as 'TOT-TCDD', meaning total tetrachlorodibenzo-p-dioxin, are appended to the list. For quantitation purposes this entry is treated as a specifically identified target. However, any results obtained for this target are regarded as tentative since its intensity may include contributions from peaks that did not match any internal or external standard. RTM can also be used to rename files, add or delete entries, or create an entire *.IRT file.

*Automatic quantitation report*

The AQR program co-ordinates data from four different files to produce the final quantitation report. One of these files will be the *.IRT file already discussed. The other three files are, the Sensitivity Parameter file (*.SP), the Quantitation Pairing file (*.QP) and the Mix Reference File (*.MRF). The *.SP file is created by the Automatic Sensitivity Calculation (ASC) program and will be discussed later. The other two files are created by the Quantitation Pairing Method (QPM) and Concentration Table Editor (CTE) programs, respectively. They are editing programs and will not be discussed.

When the AQR program is first loaded, the user enters the names of all data files required. Some sample specific data is also entered through the keyboard. These include the volume of sample injected, total volume of extract, mass or volume of the original field sample extracted, accession number, etc. Once all the data are entered and a quantitation report is requested, the data files are searched. If for any reason these files are not found, or the data read from them are incomplete, AQR stops executing and displays a message explaining the cause(s) for the delay. At this point, corrections can be made by editing any parameter or by aborting the quantitation to take some other action.

If everything is in order, the quantitation process starts by reading the *.QP file. This file lists the names of targets to be quantitated and the internal standard used for each target. A *.QP file containing the data listed in figure 5 would cause 2378-TCDD and TOT-TCDD to be quantitated by using C13-2378-TCDD as the internal standard. There are normally 25 to 35 entries in this file. The next entry is not read until the previous entry is quantitated.

Once a target and its internal standard are defined by the *.QP file, the intensity of each is found by a search for their names in the *.IRT file of the sample. The concentration of the internal standard in the original spiking solution, and the absolute response factor for the target and the quantitation standard (see below) are listed in the *.MRF and *.SP files, respectively. The corresponding values for the target and internal standard are sought by AQR in similar fashion. As a complete set of parameters needed to quantitate the target becomes available, the results are displayed on the monitor or sent to the printer, and the next entry in the *.QP file is read to begin another cycle. The resulting quantitation report

```

   Filename: Method1.QP

Entry Pairs: 2

(1) C13-2378-TCDD   FOR   2378-TCDD
(2) C13-2378-TCDD   FOR   TOT-TCDD
```

*Figure 5. Quantitation pairing file.*

contains every data file and keyboard-parameter used to quantitate the sample, and serves as a record for future reference (see figure 6). When the end of the *.QP file is reached, AQR displays or prints the names of targets that could not be quantitated and a justification. Incorrect naming of entries, which give the appearance of an absent sensitivity, or an absent concentration or intensity for the internal standard, is a frequent cause for this error. It occurs while editing or when internal standards are not completely characterized by APM or RTM.

AQR can store all quantitation results and the parameters used to obtain them (i.e. filenames and keyboard entered data) into a report file (*.RP). These files can be used later by AQR to automatically re-enter all parameters, or by the Multiple Sample Report program (MSR). With the MSR program, quantitation results from up to 200 *.RP files can be assembled and printed into a table, or into files that are compatible with commercially available programs such as Lotus 123 and Sideways.

*Other TASQ programs*

*.SP files used by AQR are created by the ASC program. This program calculates the absolute sensitivity of any specifically named peak in a *.IRT file of a standard sample. ASC can do this by reading the concentration of every standard from a *.MRF file and the volume of standard injected. For completeness, the absolute sensitivity of any entry name in the *.IRT file with the specific identifier 'TOT' is assigned a value calculated for a specifically identified entry with the same generic identifier.

Another important TASQ program is the Sequential To Random reformatting program (STR). This program does not process data. Its sole purpose is to reorganize

```

CURRENT QUANTITATION PARAMETERS ARE...                                          123456

                      STANDARD RUN FILENAME: 2ST0812.SP

                        SAMPLE RUN FILENAME: 2S0812.IRT

                           ACCESSION NUMBER: 123456

                  SPIKING STANDARD FILENAME: BATT.MRF

              QUANTITATION PAIRING FILENAME: METHOD1.QP

               AREA DETECTION LIMIT (CNTS): 100000

                  FRACTION OF SPIKED SAMPLE: 1

                     RECOVERY STANDARD NAME: C13-1234-TCDD

       CONC. OF REC. STND. SOLUTION (PG/UL): 735

  VOLUME OF REC. STND. SOL. INJECTED (UL): 3

              TOTAL VOLUME OF EXTRACT (UL): 2.9

           VOLUME OF EXTRACT INJECTED (UL): 1.1

    VOLUME OF I.S. SOULTION INJECTED (UL): 4

                  MASS OF VOLUME OF SAMPLE: 1

```

| INTERNAL STND. | NATIVE | I.S.SEN. | I.S.CNTS | I.S.CON. | NAT.SEN. | NAT.CNTS. | REC.OF I.S. | DET. LIMIT | CONCENTRATION |
|---|---|---|---|---|---|---|---|---|---|
| C13-2378-TCDD | 2378-TCDD | 2278 | 2114262 | 471 | 1946 | 11328260 | 43 | 410 pg/m2 | 14766 pg/m2 |
| C13 12378-PCDD | 12378-PCDD | 1866 | 2223329 | 471 | 2053 | 12900180 | 55 | 303 pg/m2 | 12419 pg/m2 |
| C13-123678-HXCDD | 123678-HXCDD | 1986 | 2602732 | 471 | 1928 | 5399074 | 61 | 294 pg/m2 | 5031 pg/m2 |
| C13-123678-HXCDD | 123478-HXCDD | 1986 | 2602732 | 471 | 2105 | 15272210 | 61 | 269 pg/m2 | 13040 pg/m2 |
| C13-123678-HXCDD | 123789-HXCDD | 1986 | 2602732 | 471 | 1644 | 751732 | 61 | 344 pg/m2 | 822 pg/m2 |
| C13-1234678-HPCDD | 1234678-HPCDD | 996 | 1529643 | 471 | 1912 | 6359312 | 71 | 296 pg/m2 | 5976 pg/m2 |
| C13-12346789-OCDD | 12346789-OCDD | 1133 | 2169040 | 506 | 1124 | 5455623 | 82 | 371 pg/m2 | 6418 pg/m2 |
| C13-2378-TCDD | TOT-TCDD | 2278 | 2114262 | 471 | 1946 | 74089216 | 43 | 410 pg/m2 | 96574 pg/m2 |
| C13 12378-PCDD | TOT-PCDD | 1866 | 2223329 | 471 | 2053 | 86907248 | 55 | 303 pg/m2 | 83671 pg/m2 |
| C13-123678-HXCDD | TOT-HXCDD | 1986 | 2602732 | 471 | 2105 | 45222540 | 61 | 269 pg/m2 | 38614 pg/m2 |
| C13-1234678-HPCDD | TOT-HPCDD | 996 | 1529643 | 471 | 1912 | 15230860 | 71 | 296 pg/m2 | 14311 pg/m2 |

*Figure 6. Quantitation report.*

integrated data files so they can be accessed by the APM program as random files. Data can be found and read faster from a random file.

The remaining TASQ programs can create relative retention time tables from *.IRT files (Relative Retention Time, RRT), average relative retention times from several *.IRT files (Retention Time Statistics, RTS), tabulate absolute and relative response factors defined by a *.QP file and using a *.SP file or averaging several *.SP files (Response Factor Report, RFR), allow the user to examine integrated data files (Integration File Window, IFW), etc.

*TASQ enhancements*

As the user alternates from among 14 different programs, keeping track of data processing events becomes difficult. To lessen this effect, menu-driven features were incorporated that allow loading programs by depressing a single function key. After a program is executed, the menu is automatically restored. Useful information appearing on the menu include the last program loaded, the last file opened, input files required and output files created by all programs, and documentation keys explaining the use and purpose of each program.

Tandem processing of data files is possible when using STR, APM, RFR, RTS or MSR. These programs can read files containing a list of filenames to be used for input. Filename directories are easily created by using PC-DOS commands or any suitable ASCII editor.

### Results

*Speed of analysis*

Although some degree of automation was possible before TASQ was implemented, the time saved by combining automated and manual data processing of integrated data files was not significant. Thus, the immediate rewards associated with replacing a labor-intensive procedure with one that is totally automated by a microcomputer, are obvious. Table 1 shows the average processing times determined for the three basic TASQ programs: APM, RTM and AQR. Also shown are the average times required to transfer files from the HP-workstation to the IBM XT, and to reformat integrated data files using the STR program. These results do not take into account the added convenience of dedicating the GC/MS system to almost full time data acquisition. In addition, with the arrival of 80286 and 80386 processor based PCs, and faster hard disk drives, faster processing speeds are expected.

*Table 1. Average Processing Times (APT).*

| Program or Process | Function | APT (sec./file) | Notes |
|---|---|---|---|
| Transferring data files | | 91 | a |
| STR | Formatting files | 22 | b |
| APM | Peak matching | 39 | c |
| RTM | Internal-RTM | <3 | d |
| RTM | External-RTM | <3 | d |
| RTM | Totalling | <5 | d |
| AQR | Quantitate | 30 | e |

(a) Using Kermit 2·29, Baud Rate: 4800.
(b) Average input file size: 45 kbytes.
(c) Average input file size: 65 kbytes.
(d) Average number of entries per file: 90, including 12 internal standards.
(e) A printed report of 46 quantitations.

Assembling tables of quantitation results can be easily accomplished with the help of the MSR program. In a recent study in which PCDFs and PCDDs from stack emissions were monitored, 30 targets were quantitated in 93 samples. Each target quantitation consisted of three results; the percent recovery of internal standard, the target detection limit and the target concentration. To prepare a report for this study, a table was constructed by sorting 8370 quantitation results by sample accession number and target name. A task that would have normally taken several hours was accomplished in about 2 min of data-processing with the MSR program, and 10 min of printing a text file (produced by MSR) using Sideways.

*Data reporting*

Routine analysis for PCDFs and PCDDs is often performed at the request of government of public health agencies. Thus, quantitation results may be used for the purpose of establishing environmental policies, assessing human risk, or determining a course of legal action. In light of these facts, it is essential to keep accurate records documenting every phase of sample analysis to ensure the validation of quantitation results.

A number of TASQ programs can produce files or reports which allow closer inspection of data and data-processing events. For example, each time the APM program is used a file is created that contains a detailed account of the peak matching process. The printed version of this report is kept with all quantitation records and, when necessary, it is used to trace the decision-making path followed to characterize peaks as non-targets or targets. When standards or spiked samples are analysed, this feature is particularly useful for evaluating instrument performance and fine-tuning peak matching parameters. Figure 7 shows an example of part of this 'peak matching report'.

```
TARGET: TCDF

QUAN. MASS: 306            RATIO MASS: 304

-------------------------------------------

R.T.     INTENSITY        R.T.     INTENSITY

-------------------------------------------

35.935   385496* *        35.936   286312* *

36.457   233493* *        36.489   170587* *

38.200   182163*          38.172    57489*

38.504   124164            0.000        0

-------------------------------------------

Legend:

   [* *] intensity and retention time match

    [*]  retention time match only

    [ ]  no matches

```

*Figure 7. Peak matching report.*

Retention time and response factor averaging are other TASQ features that are also useful in this area.

*Other applications*

The criteria methods by which PCDDs and PCDFs are identified and quantitated from GC/MS data are applicable to a variety of other important toxic environmental contaminants of interest. TASQ quantitations have been applied to polychlorinated biphenyls (PCBs), chlorinated and brominated benzenes, and hexachlorinated cyclohexanes with similar success. In theory, any target which can be identified by GC/MS on the basis of matching retention times and intensity ratios is suitable for TASQ processing.

While all files generated by TASQ programs are in ASCII format, many popular scientific, spreadsheet and word processing programs can easily import files of this type. As already mentioned, the MSR program can produce files that are compatible with commercially available software. In the case of Lotus 1, 2, 3, entire quantitation tables can be transferred, allowing the user to take advantage of the many data-processing features the spreadsheet has to offer. Similar links are being established with PlotIT (Interactive Plotting and Statistics Package) [9] and SIMCA (Soft Independent Modeling of Class Analogy) [10] software. In the latter case, pattern recognition techniques are being applied to investigate a possible relation between the concentration profile of samples and sources of contamination.

*Adaptability*

Since TASQ was created, three updated software versions from Hewlett-Packard have been used. On each occasion, integrated data files were formatted differently and APM's file searching routines had to be modified. However, modifying and compiling APM's source code was easily accomplished in less than one hour. No other programs had to be changed since they do not use integrated data files, only files created by APM and thereafter.

To this date, TASQ has been used to process only data files from the HP data system. However, by modifying the APM program, it is possible to use data files from any source capable of transferring data to a printer port. The feasibility of using files from a Kratos MS-50 GC/MS system is currently being studied. If successful, it would make possible the processing of data from high mass resolution acquisitions.

*Costs*

Considering the expense of staffing and equipping a laboratory for environmental analysis, the additional costs of implementing this system are minimal. The bulk of all material expenses is the purchase price of a microcomputer. These units are available for less than $2000. The cost for generating the software is estimated at approximately $15 000. However, release of a commercial version of TASQ is being considered by the New York State Department of Health and Health Research Incorporated.

### Conclusions

External data processing of integrated data files from a Hewlett-Packard Gas Chromatograph/Mass Selective Detector system, has made it possible to totally automate a formerly manual processing scheme, resulting in a notable increase in productivity. The higher processing speed, convenience of dedicating the GC/MS system to nearly full time data acquisition, ability to quantitate targets for which no standards are available, and the ease with which reports and tables can be generated, have all contributed to this result. Furthermore, the ability to automatically transfer quantitation results to programs such as Lotus 1, 2, 3, provides the user with a variety of data-processing options previously not available. Finally, given the high cost of environmental analysis, the expense incurred by implementing this processing scheme is minimal when compared to the savings in man-hours, and instrument time, which in turn reduces the overall cost of analysis.

### Acknowledgements

The author wishes to thank Drs Kenneth M. Aldous, Robert G. Briggs and David R. Hilker, at the New York State Department of Health, for linking the Hewlett-Packard system to the IBM XT and helpful discussions concerning BASIC programming techniques, and Kathleen Harris-Valente for reviewing the text for clarity and accuracy.

### References

1. Slivon, L. E., Gebhart, J. E., Hayes, T. L., Alford-Stevens, A. L. and Budde, W. L., *Analytical Chemistry*, **57** (1985), 2464.
2. Hein, C. S., Gill, J. P., Evershed, R. P. and Eglinton, G., *Analytical Chemistry*, **57** (1985), 1872.
3. McFadden, W. H., in *Techniques of Combined Gas Chromatography/Mass Spectrometry: Applications in Organic Analysis*, Wiley Interscience (New York, 1973), and references cited therein.
4. McLafferty, F. W., in *Interpretation of Mass Spectra*, University Science Books (Mill Valley, CA, 1980), p. 10.
5. ASCII, American Standard Code for Information Interchange.
6. KERMIT, Version 2.20 by Daphne, T. *et al.* (Columbia University).
7. Lotus 1, 2, 3, Release 2, Lotus Development Corporation (Cambridge, MA, 1985).
8. Sideways, Version 2.01, Funk Software Inc. (Cambridge, MA, 1982).
9. PlotIT, by Eisensmith, S.P., Scientific Programming Enterprises (Haslett, MI, 1985).
10. SIMCA-3B, Version 821112, Principal Data Components (Columbia, MO, 1985).

